# Resident perception of Obstetrics and Gynecology(OBGYN) residency pelvic anatomy curriculum: a national study

**DOI:** 10.1186/s12909-025-07485-0

**Published:** 2025-07-23

**Authors:** Sharonne Holtzman, Lily McCarthy, Isabel S. Chess, Yara Sifri, Farida Nentin, Frederick Friedman

**Affiliations:** Icahn School of Medicine at Mount Sinai, Department of Obstetrics, Gynecology and Reproductive Medicine, New York, NY USA

**Keywords:** OBGYN resident education, Pelvic anatomy, Resident curriculum

## Abstract

**Background:**

Pelvic Anatomy is an integral part of the educational objectives in Obstetrics and Gynecology (OBGYN) residency. As a surgical subspecialty, mastering pelvic anatomy is imperative in performing successful surgical procedures, reducing surgical complications, and improving surgical outcomes. The objective of this study was to conduct a national survey to examine the OBGYN resident perspective on their residency’s pelvic anatomy curriculum.

**Methods:**

After Institutional Review Board at the Mount Sinai Hospital approval, an 18-question survey was distributed to all 241 ACGME program coordinators to distribute to their residents. The survey was circulated electronically using SurveyMonkey tool and on four different dates including: 11/18/2021, 2/24/2022, 4/11/22, and 5/2/22. All demographic information, training environment and perspective on pelvic anatomy was collected anonymous through the survey. All statistical analysis was done using SPSS 2.0.

**Results:**

Out of 241 programs, we received responses from 28 programs with a total of 582 possible residents in their respective programs. A total of 180 residents responded to our survey which is a 31.0% response rate. Out of all the residents, 46 (25.84%) were PGY1, 40 (22.47%) were PGY2, 43 (24.16%) were PGY3 and 49 (27.53%) were PGY4. The majority of residents, 172 (95.5%), were between the ages 25–34, and 155 (86.1%) identified as female. The majority, 125 (69.4%), of residency programs were considered academic and 99 (55.0%) of the respondents considered the location of their residency as urban. All ACOG districts were represented in our survey.

Throughout their residency training, 104 (58.10%) residents experienced formal education on pelvic anatomy in their residency and 75 (41.90%) did not. In terms of surgical curriculum, 138 (77.53%) participated in a formal surgical simulation curriculum and 40 (22.47%) did not. There were 159 (88.83%) who had protected lecture time that focused on pelvic surgical anatomy, but only 100 (62.9%) residents felt that these lectures were helpful for their education. When residents were asked if they felt that their OBGYN program should have formal time in an anatomy lab from a scale of 1–10, the average answer was 7.5. When the residents were asked if they felt that formal training in anatomy lab would help them become a better surgeon, the average answer was 9.0. For our primary outcome, when ranking their own program from a scale of 1–10, residents felt that their program trained them in all aspects of OBGYN on average of an 8. On multivariable analysis, residents who had formal education on pelvic anatomy as well as those residents who had a surgical simulation curriculum as part of their OBGYN residency felt that their residency program was more likely to adequately train them in all aspects of OBGYN (*p* < 0.05).

**Conclusions:**

Pelvic anatomy education is an important topic for residents during their OBGYN training. Only 60% of programs have a formal pelvic anatomy curriculum and 89% had formal lectures on pelvic anatomy, with majority of residents finding pelvic anatomy lectures to be unhelpful for their education. Residency programs should consider adding hands-on, formal anatomy education to their curriculum to adequately train their residents in the field of OBGYN.

## Background

Obstetrics and gynecology (OBGYN) residency plays a critical role in shaping the knowledge and skills of future OBGYN practitioners. Proficiency in pelvic anatomy does not only facilitate successful performance of surgical procedures but also contributes to minimizing surgical complications and optimizing surgical outcomes [1]. In the 12th edition of the educational objectives of the Council on Resident Education in Obstetrics and Gynecology (CREOG), it states that Pelvic Anatomy is an integral part of the educational objectives during OBGYN residency [2]*. Specifically, as surgical subspecialists, an in-depth understanding of pelvic anatomy is imperative for surgical success.

Despite the acknowledged importance of pelvic anatomy education, there is considerable variation among OBGYN residency programs in terms of the curriculum and educational approaches employed. The diversity in educational strategies includes didactic lectures, surgical simulation, and hands-on dissection [3]*. This variety raises questions about the effectiveness and comprehensiveness of current training methodologies and which method is most effect for the training of the specific OBGYN trainee.

To our knowledge, this is the first study examining the perspective of OBGYN residents on their pelvic anatomy and surgical curriculum. The objective of this study was to conduct a national survey to examine the OBGYN resident perspective on their residency’s pelvic anatomy curriculum.

## Methods

After this study was approved by the Institutional Review Board at the Icahn School of Medicine at Mount Sinai, an 18 question survey was distributed to all 241 ACGME OBGYN residency programs in the United States. The survey was circulated electronically using SurveyMonkey® tool and on four different dates including: 11/18/2021, 2/24/2022, 4/11/22, and 5/2/22. The survey was distributed to each program coordinator and director, with attempts to find the most recent and up-to-date email address. The survey responses were anonymous and no identifiable information was linked to survey responses, including names, IP addresses, and emails. The survey response rate was calculated based on response by residency program with confirmation of distribution of survey to their respective residents. The number of potential responses was calculated based on the known number of residents in each program which confirmed distribution of the survey.

Along with demographic questions, 9-questions were developed with 5 multiple-choice questions and 4 questions requiring a response on a scale of 1–10. Demographic and training information collected included age, post-graduate training year, gender identity, fellowship aspirations, training environment, geographic environment, ACOG district, and number of residents in each program. All responses that were used in analysis were based on self-reported data. The survey underwent a pilot test by five recent OBGYN residency graduates to evaluate clarity of questions, overall coherence and time of survey completion. The primary outcome of this study was the OBGYN residents’ perspective on their general training, specifically in regards to pelvic anatomy and surgical curriculum. This was assessed on a scale of 1–10 by four different questions in the questionnaire.

Demographics and characteristics of respondents are reported descriptively as mean and standard deviations (SD) for continuous variables or frequencies and proportions for categorical measures. Descriptive statistics, paired-samples t tests, and multivariate linear regression with stepwise modeling were performed using IBM SPSS 2.0. Statistical significance was determined at *p* < 0.05.

## Results

Out of 241 programs, we received responses from 28 programs with a total of 582 possible residents in their respective programs. A total of 180 residents responded to our survey which is a 31.0% response rate. Out of all the residents, 46 (25.84%) were PGY1, 40 (22.47%) were PGY2, 43 (24.16%) were PGY3 and 49 (27.53%) were PGY4. The majority of residents, 172 (95.5%), were between the ages 25–34 and 155 (86.1%) identified as female. Most (125 (69.4%)) of residency programs were considered academic and 99 (55.0%) of the respondents considered the location of their residency as urban. All ACOG districts were represented in our survey (Table 1).

**Table 1:**
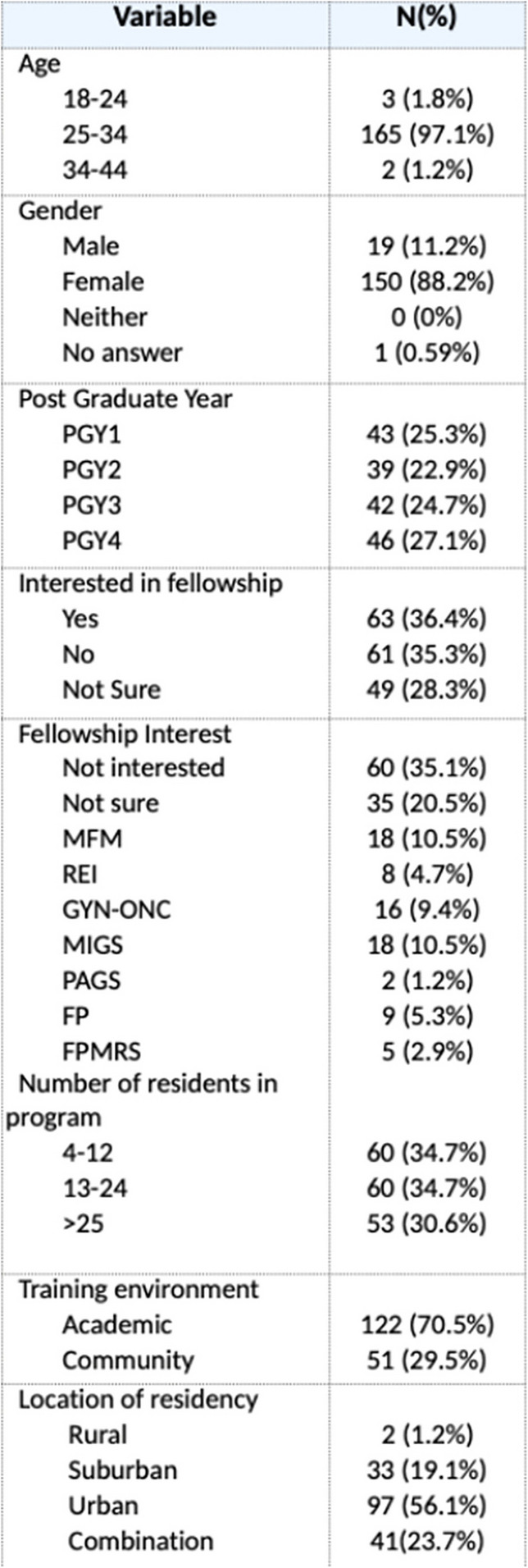
Resident demographics

Out of all residents, 104 (58.10%) experienced formal education on pelvic anatomy in their residency and 75 (41.90%) did not. In terms of surgical curriculum, 138 (77.53%) participated in a formal surgical simulation curriculum and 40 (22.47%) did not. There were 159 (88.83%) who had protected lecture time that focused on pelvic surgical anatomy, yet only 100 (62.9%) residents felt that these lectures were helpful for their education. On a scale of 1–10, the average response was 7.5 when residents were asked if they felt that OBGYN program should have formal time in an anatomy lab and 9.0 when residents were asked if they felt that formal training in anatomy lab would help them become a better surgeon. To the question of how strongly anatomy is best learned in the operating room on a scale of 1–10, the average score was 8.5. The responses to these questions did not differ based on demographic characteristics of the trainee including age, gender, or post-graduate year (*p* > 0.05).

**Fig. 1:**
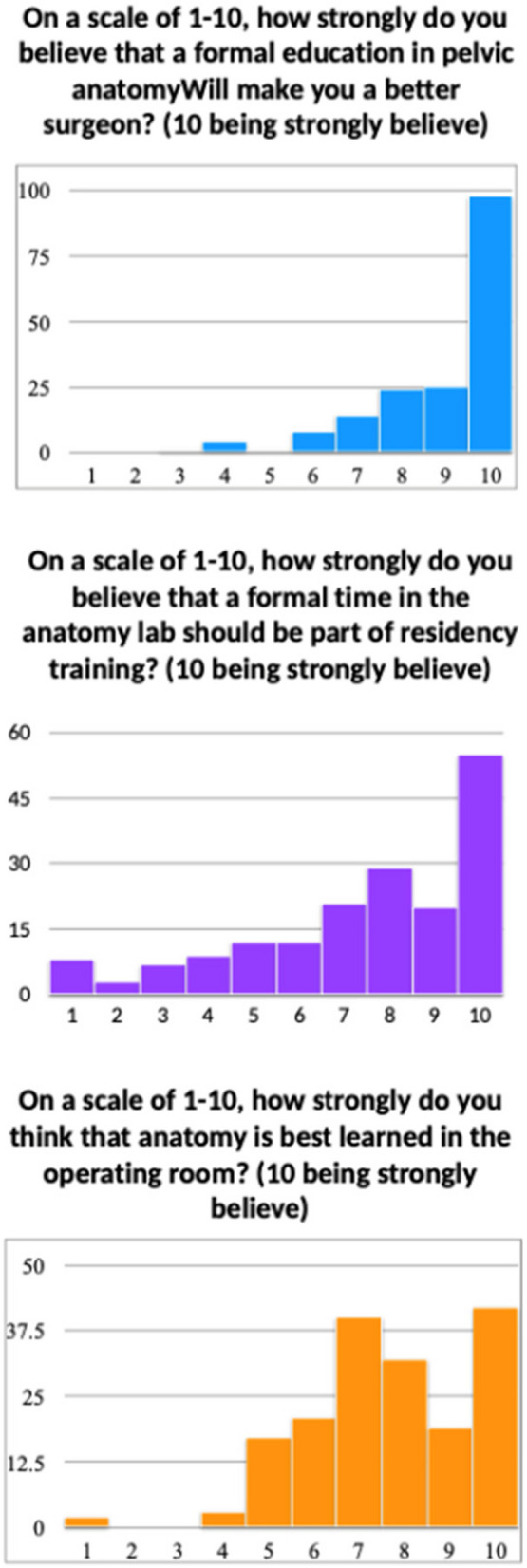
Resident interest in formal education in pelvic anatomy

**Fig. 2:**
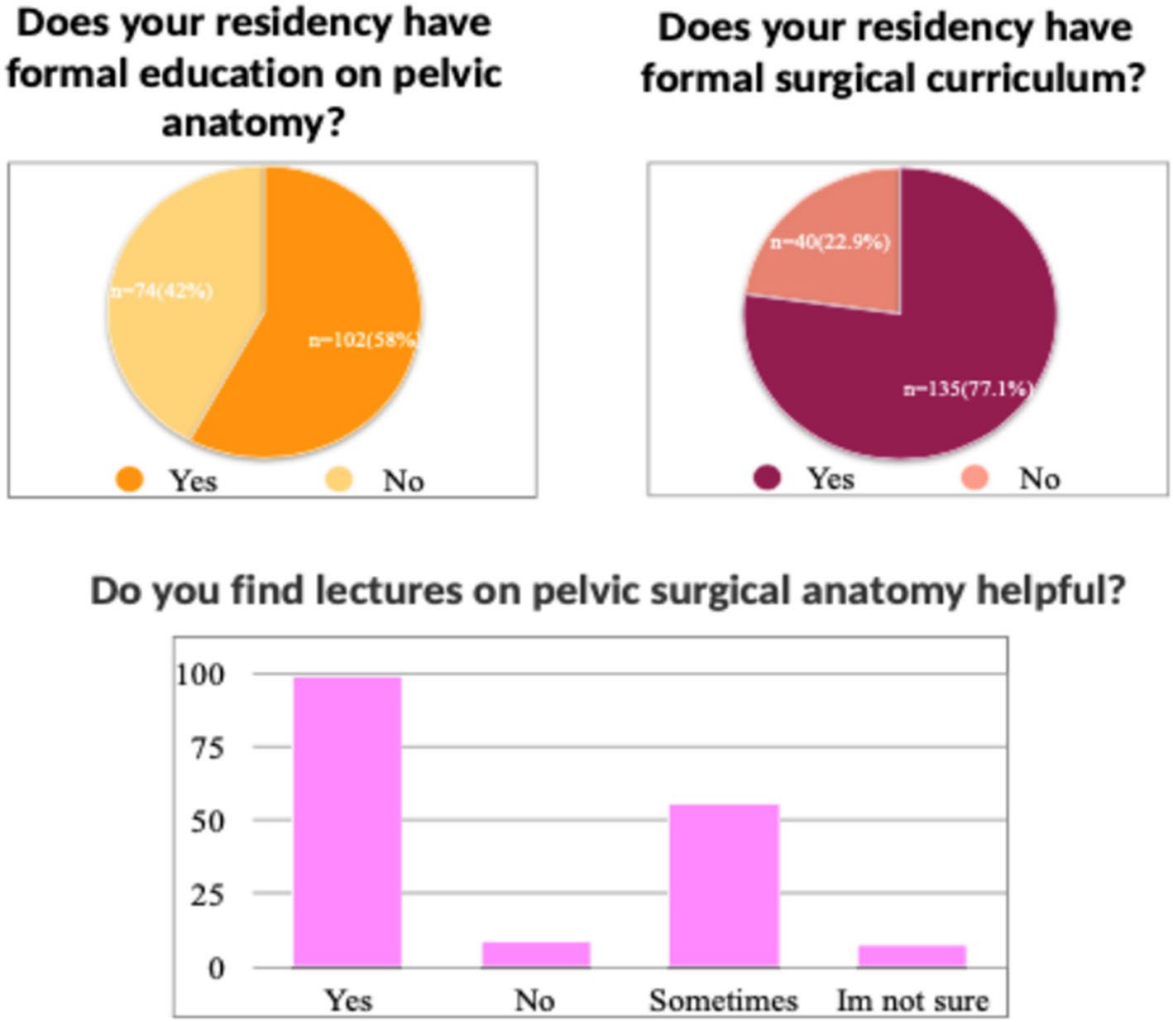
Resident formal pelvic anatomy education access

When ranking their own program from a scale of 1–10, residents felt that their program trained them in all aspects of Obstetrics and Gynecology on average of an 8. On multivariable analysis, residents who had formal education on pelvic anatomy as well as those residents who had a surgical simulation curriculum as part of their OBGYN residency felt that their residency program was more likely to adequately train them in all aspects of OBGYN (*p* < 0.05) (Figs. 1 and 2).

## Discussion

This study emphasizes the importance of hands-on approach in formal training for pelvic anatomy for residents. To our knowledge, the current study is the first to date to explore OBGYN residents’ perspectives on pelvic anatomy education in this degree of depth. The lack of literature on pelvic anatomy education in OBGYN residency is somewhat surprising given the centrality of anatomy in medical education overall and in the field of OBGYN in particular [2]*. Pelvic anatomy is considered to be foundational to comprehension of pelvic surgery, in addition to other aspects of OBGYN such as labor and delivery [2]*. Sophisticated understanding of pelvic anatomy is regarded as vital in surgeries for common gynecological problems including pelvic organ prolapse, which necessitate substantial anatomical knowledge and technical expertise in order to conduct safe dissections and reduce complications such as ureteral injury [3, 4]. Core pelvic surgeries such as hysterectomies can pose a number of challenges to OBGYN residents who are at early stages of their training [5].

Despite the dearth of data on pelvic anatomy education, there have been a few studies that have explored this topic and reported findings similar to ours. Mizrahi et. al. administered a small single-center survey on educational and clinical objectives in pelvic anatomy to quantify 52 OBGYN residents’ knowledge of pelvic anatomy and found that most of these residents (92%) were dissatisfied with the anatomical teaching they had received and expressed interest in learning through interactive anatomy videos rather than more traditional anatomy lectures and textbooks [2]*. This finding aligns with our own, as we similarly found that a sizeable percentage of residents felt underwhelmed by their respective institutions’ approaches to pelvic anatomy education.

Other investigations have identified a similar trend of disappointment expressed by residents in the caliber of pelvic anatomy education provided by residency programs [6]. These include one by Sgroi et. al., who found that an overwhelming 84% of residents in the Royal Australian and New Zealand College of Obstetricians and Gynecologists Interated and Elective Training Program viewed the pelvic anatomy teaching offered by the program as insufficient [7]. In a separate survey-based study by Cornet et. al., residents also expressed a need for more instruction in pelvic floor anatomy and training specifically in the area of perineal tear repair, with the majority of residents (62%) in the cohort characterizing their grasp on pelvic anatomy as poor [8]. Thus, there is clearly a sentiment shared by a wide range of residents that there are significant knowledge gaps in pelvic anatomy and that current curriculums may leave much to be desired.

### Clinical implications

The results from this study, specifically our finding that residents who underwent both formal education on pelvic anatomy and training through a surgical simulation curriculum felt better prepared by their institution in every dimension of OBGYN, add to the growing body of literature supporting the benefits of pelvic anatomy education in OBGYN residency training. An increasing number of studies have demonstrated that comprehensive pelvic anatomy and dissection courses are associated with significant improvements in OBGYN residents’ level of overall comfort, anatomical knowledge, and technical competency [9–12].

For instance, in a survey study by Tjalama et. al., a group of residents who participated in a laparoscopic training course designed to expand anatomical knowledge and technical skills, involving lectures on relevant pelvic anatomy, multiple operative videos, and hands-on cadaveric dissections [13]. After completion of the course, the majority of participants gave very positive feedback and viewed the education and exercises as highly valuable for future surgical endeavors [13].

Another study by Chong et. al., investigated the influence of various forms of instruction on transobturator vaginal tape insertion on 34 OBGYN residents’ anatomical expertise and surgical prowess, ultimately finding that participants’ confidence and knowledge scores improved substantially following cadaver dissection and pelvic module simulation [15]. Interestingly, transobturator vaginal tape insertion scores were significantly higher in residents who trained with cadavers than those who used a bony pelvis and instructional videos (*p* < 0.01), suggesting that hands-on training may be an even more effective educational strategy [15]. Additional studies have examined the efficacy of other educational tactics including clay pelvic models, which likewise resulted in higher self-reported confidence, knowledge, and satisfaction among residents involved [16, 17].

Our findings provide further insight into the value of proper pelvic anatomy education and its potential to strengthen the quality of OBGYN training, which holds relevance for clinicians and medical education authorities alike. The results from this study reveal that there is indeed a perceived knowledge gap in pelvic anatomy education in OBGYN residency despite its clear utility in common clinical and surgical contexts, both of which were affirmed by many of the residents queried.

### Research implications

This study is the first of its kind to investigate residents’ perspectives on the current state and potential areas for improvement in pelvic anatomy education across multiple institutions, levels of training (i.e. PGY1 through PGY4), and knowledge and training domains. However, there remains a need for larger-scale studies involving much greater cohorts to fully address the place of pelvic anatomy education in resident program curricula, which future investigations will doubtlessly clarify. In addition, although this question was outside the scope of the present study, the optimal method and mode of delivery of pelvic anatomy instruction remains unclear; this is the case despite increasing indications that hands-on learning with real-life clinical applications and technologies may be the best approach for boosting residents’ sense of confidence and competence. While this study examines resident perspectives, it is possible that the mode of delivery is not as much of an issue as the context and ability to apply the knowledge. Finally, even though this study included several graded scaling systems and statistical analysis, our examination of residents’ points of view was mostly qualitative in nature and did not answer quantitative questions such as the extent to which receiving different types of instruction in pelvic anatomy correlated with computable increases in anatomical understanding. In order to determine if educational activities are genuinely useful and effective, future studies should evaluate their effects on residents’ proficiency in real clinical settings such as the operating room [14].

### Strengths and limitations

This study has several strengths, including the wide representation of every ACOG district in the survey we administered. The demographic distribution of survey participants is also another strong point of our investigation. For example, there was a relatively equivalent split of residents from different training locations, and there was also a similar number of residents in each of the 4 years of OBGYN residency surveyed. Our specific focus on resident education in particular also represents another asset of the current study. Several prior studies have examined pelvic anatomy education for individuals at different stages of their training, such as medical school students [18–20] and OBGYN residents as compared to urology residents [21]. The fact that we only surveyed residents makes our findings specifically relevant to this particular population of trainees and may therefore be easier to translate directly into resident education.

Still, this study also has a few weaknesses that limit its generalizability. These include the relatively small response rate (31%), which may not necessarily capture the full range of resident responses. The conclusion in our study reflect the residents who responded to our survey. In addition, the fact that it is a survey-based study makes it inherently subjective. Unfortunately, it is challenging to compare the weaknesses of our present study to those from other studies, and to determine if there are any significant differences in reported data, given how scant literature is on this topic. Future work will likely address many of these concerns and shine further light on the potential import and clinical implications of pelvic anatomy education in OBGYN residency. Future directions could include surveying program directors to understand why pelvic anatomy education in residency may not be as helpful as expected to enhance our understanding of how to improve pelvic anatomy education.

## Conclusions

In conclusion, this study contributes to the growing body of evidence supporting the integration of pelvic anatomy education into OBGYN residency curricula. Despite the recognized value of such education, only slightly more than half of the residents in our study had received formal instruction in this area. Among those who did receive official pelvic anatomy lectures, the majority from them found the lectures unhelpful for clinical practice. However, residents who had combined formal pelvic anatomy education with surgical simulation training reported feeling better prepared in all aspects of OBGYN. These findings suggest that residency programs should consider incorporating hands-on teaching of pelvic anatomy to enhance residents’ knowledge and technical skills.

## Data Availability

The datasets used and/or analyzed during the current study are available from the corresponding author on reasonable request.
